# Spatial localization during open-loop smooth pursuit

**DOI:** 10.3389/fnins.2023.1058340

**Published:** 2023-02-02

**Authors:** Stefan Dowiasch, Marius Blanke, Jonas Knöll, Frank Bremmer

**Affiliations:** ^1^Department Neurophysics, Philipps-Universität Marburg, Marburg, Germany; ^2^Center for Mind, Brain and Behavior (CMBB), Philipps-Universität Marburg and Justus–Liebig–Universität Gießen, Gießen, Germany; ^3^Institute of Animal Welfare and Animal Husbandry, Friedrich-Loeffler-Institut, Celle, Germany

**Keywords:** smooth eye movements, smooth pursuit, localization, open-loop eye movement, open-loop SPEM, localization error

## Abstract

**Introduction:**

Numerous previous studies have shown that eye movements induce errors in the localization of briefly flashed stimuli. Remarkably, the error pattern is indicative of the underlying eye movement and the exact experimental condition. For smooth pursuit eye movements (SPEM) and the slow phase of the optokinetic nystagmus (OKN), perceived stimulus locations are shifted in the direction of the ongoing eye movement, with a hemifield asymmetry observed only during SPEM. During the slow phases of the optokinetic afternystagmus (OKAN), however, the error pattern can be described as a perceptual expansion of space. Different from SPEM and OKN, the OKAN is an open-loop eye movement.

**Methods:**

Visually guided smooth pursuit can be transformed into an open–loop eye movement by briefly blanking the pursuit target (gap). Here, we examined flash localization during open-loop pursuit and asked, whether localization is also prone to errors and whether these are similar to those found during SPEM or during OKAN. Human subjects tracked a pursuit target. In half of the trials, the target was extinguished for 300 ms (gap) during the steady–state, inducing open–loop pursuit. Flashes were presented during this gap or during steady–state (closed–loop) pursuit.

**Results:**

In both conditions, perceived flash locations were shifted in the direction of the eye movement. The overall error pattern was very similar with error size being slightly smaller in the gap condition. The differences between errors in the open- and closed-loop conditions were largest in the central visual field and smallest in the periphery.

**Discussion:**

We discuss the findings in light of the neural substrates driving the different forms of eye movements.

## Introduction

The localization of visual targets is of critical importance in everyday life. Accurate localization is a challenging task, however, as eye movements constantly change the image of the outside world that falls onto the retina. Nevertheless, we perceive the outer world as stable. Yet, different from introspection, this stability is not complete. Many studies have shown that targets, which are presented briefly (flashed) during eye movements, are systematically mislocalized. The pattern of localization error depends on the type of eye movement and the exact experimental conditions.

Even during active fixation which is a distinctive class of eye movements (Carpenter, 1988; Rucci and Poletti, 2015; Rucci and Victor, 2015; Krauzlis et al., 2017) localization is not accurate. Numerous studies have found localization errors during fixation. The robustness of this effect can be deduced from the fact that it was found with a broad range of response modes [ruler with (random) numbers or letters: Mateeff and Gourevich, 1983; Kaminiarz et al., 2007; mouse pointing: Sheth and Shimojo, 2001; (verbal) report: Hafed, 2013; cursor movement and button press: Willeke et al., 2022]. During smooth pursuit, i.e., a voluntary eye movement serving to keep the image of a moving object of interest in the fovea, the perceived location of briefly flashed targets is reported to be shifted in the direction of the eye movement. This perceptual shift, or localization error, is stronger in the hemifield the eye is heading toward (ipsiversive hemifield) than in the opposite, contraversive hemifield. Like for the studies investigating spatial perception during fixation, also this effect is quite robust, as indicated by various response measures [ruler with (random) numbers: Mitrani and Dimitrov, 1982; Klingenhoefer and Bremmer, 2009; Koenigs and Bremmer, 2010; finger tapping: Rotman et al., 2004; relative localization with respect to continuously shown reference points: van Beers et al., 2001; saccades as well as mouse pointing: Blohm et al., 2003]. During pursuit initiation, i.e., the transition from active fixation to active tracking of a moving target, a change in spatial localization from fixation-like to pursuit-like is observed already for flashes presented well before the eyes start to move, as tested by localization with respect to continuously shown reference stimuli (Blanke et al., 2010). Localization errors also occur during saccades, i.e., the fast ballistic eye movements that aim to bring a target of interest into the fovea. The pattern of localization error critically depends on the exact experimental conditions, varying between a shift and a compression of perceptual space (Honda, 1989; Cai et al., 1997; Ross et al., 1997; Kaiser and Lappe, 2004). Here, the term *shift* refers to the finding that the perceived positions of flashes across the whole visual field are reported to be shifted by the same amount and in the direction of the saccade. The term *compression*, on the other hand, refers to the finding that flash positions across the whole visual field are perceived closer toward the saccade target than they actually are.

Optokinetic nystagmus (OKN) is a reflexive eye movement induced by large field visual motion. It consists of alternating phases of slow and fast eye movements. The fast phases share some characteristics with saccades (Kaminiarz et al., 2009), whereas slow phases look like smooth pursuit eye movements (Carpenter, 1988). Also, during slow-phase OKN flashed targets are mislocalized in the direction of the eye movement. Different from the localization error observed during smooth pursuit, however, the localization error during OKN is rather constant across the visual field, as evidenced by various response modes (ruler with random numbers: Kaminiarz et al., 2007; verbal report: Tozzi et al., 2007). After prolonged performance of OKN the alternation of slow and fast phases is continued in the absence of visual stimulation (Carpenter, 1988). This eye-movement pattern is called optokinetic after-nystagmus (OKAN). While flashed stimuli are also mislocalized during the slow phase of OKAN, the error pattern is very different from that observed for OKN: targets flashed during OKAN are perceived as being more eccentric than they really are, leading to a perceived expansion of visual space, independent of the eye-movement direction (Kaminiarz et al., 2008). OKAN is considered a so-called *open-loop* eye movement. For smooth pursuit, an open-loop condition can be induced by temporarily occluding the pursuit target (gap). During this open-loop smooth-pursuit phase, eye velocity starts to drop after around 190 ms, yet it does not decay completely. After around 280 ms, the velocity stabilizes to what has been termed residual velocity, which is related to, yet lower than, pre-occlusion pursuit velocity (Becker and Fuchs, 1985). Importantly, this gap-induced open-loop condition during steady-state pursuit is different from open-loop during pursuit initiation. As shown recently, visual signals can in principle affect pursuit initiation (Buonocore et al., 2019). Open loop in the steady-state (i.e., gap) condition, however, is characterized by a missing visual foveal target, and hence qualitatively similar to the optokinetic afternystagmus (OKAN).

In our current study, we aimed (i) to determine localization during steady-state open-loop pursuit and (ii) to compare it to localization during the other slow eye movements (closed-loop pursuit, slow-phase OKN and OKAN). More specifically, we wanted to determine, whether localization error is similar to slow-phase OKAN or closed-loop smooth pursuit, since both have comparable oculomotor parameters but differ in terms of the presence/absence of an explicit visual target. We thus measured localization of flashed stimuli during continuous presentation of the pursuit target (continuous condition) and during transient occlusion of the pursuit target (gap condition) in an otherwise identical paradigm.

## Materials and methods

### Subjects

Subjects were eight healthy adults, (three male, five female; age 21–29) with normal or corrected to normal vision. One subject is author (MB) while the other seven subjects were naïve with respect to the purpose of the study. Four subjects had prior experience with eye-movement studies. All subjects gave informed written consent prior to the experiment and all procedures conformed to the Declaration of Helsinki. Experiments were carried out in complete darkness in a sound attenuated room. Computer generated stimuli were projected onto a large tangent screen (114 cm viewing distance, spanning 70° by 55° visual angle) by a CRT projector (Marquee 8000, Electrohome Inc., Kitchener, Ontario, Canada; 1,152 × 864 pixels spatial resolution, 100 Hz frame rate). Eye positions were recorded using a head mounted infrared eye tracker (EyeLink II, SR Research, Canada; 500 Hz sampling rate) and stored on disk for offline analysis.

Measurements were performed in blocks of trials. One block consisted of 100 trials and lasted 10–13 min. On average, data acquisition per subject consisted of about 50 blocks. Subjects determined themselves when to take a break to maintain their ability to concentrate, which they typically did after two to four blocks. The experimenter rarely suggested breaks based on subjective criteria (increased frequency and duration of blinks). Prior to each block the eye-tracking system was calibrated via a nine-point grid (three by three). Every ten trials a gray screen with a central fixation point for *drift correction* was presented. The subjects completed up to nine blocks during a single recording session, which lasted between 1 and 3 h, including breaks. Recording sessions were separated by at least 4 h, usually only one (at maximum two) recording session was performed per day.

### Pursuit paradigm

**FIGURE 1:**
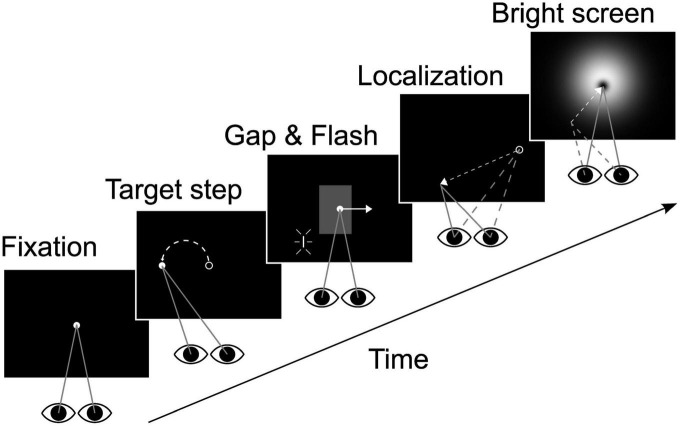
Experimental design. After initial fixation (500 ms) the target was displaced by 12° either to the left or right (only leftward displacement shown here) and immediately started to move into the opposite direction at 10°/s (2,400 ms pursuit duration). The localization target (“flash”) was presented when the eyes were in the center of the screen. In one half of the trials the target was temporarily blanked (“gap” area indicated in gray in the middle panel, invisible in real trials). Blanking started 200 ms before and lasted till 100 ms after flash presentation. When the target reached 12° eccentricity it was extinguished and subjects performed a localization saccade to the perceived flash position. After position confirmation by key stroke a bright screen was presented to prevent dark adaptation. Flash or eye positions in the individual panels are not drawn to scale. See main text for geometrical details.

The experimental design is depicted schematically in Figure 1. Participants initially fixated on a Gaussian blob (σ = 0.3°, peak luminance 12 cd/m^2^, subjective size ∼0.5°) presented for 500 ms at eye level in the middle of the tangent screen. The target then was displaced horizontally by 12° to the left or right from where it instantaneously started moving for 2,400 ms at 10°/s in the opposite direction, i.e., leftward for a rightward step and *vice versa* (step-ramp stimulus). Our paradigm had two visibility conditions: in one half of the trials the target was continuously visible for the entire time of motion (closed loop or *continuous condition*). In the other half the target was blanked from −2 to +1° horizontal eccentricity (relative to straight ahead) for rightward pursuit and +2 to −1° for leftward pursuit (open loop or *gap condition*). Regardless of the visibility condition a localization target (“flash,” a vertically oriented line of 0.25° width and 2° height) was flashed for one video-frame (decay of the phosphors: τ = 2 ms) when the pursuit target crossed the center of the screen. The choice of parameters for blanking was guided by two concerns: (i) maintenance of saccade-free pursuit and (ii) avoiding putative effects of target offset on localization of the flash. Thus, we set the occlusion duration to 300 ms and the time interval between pursuit target disappearance and flash to 200 ms. The center of the flash was placed 2° beneath the horizontal meridian at one of five horizontal eccentricities (−8°, −4°, 0°, +4°, and +8°, relative to the center of the screen). The 20 different conditions (two visibility conditions * two pursuit directions * five flash positions) were presented in pseudo-randomized order to minimize any anticipatory effects.

Subjects were informed that the pursuit target would always reappear shortly after disappearing. They were instructed to track the moving target regardless of visibility and to remember the location of the flash. Subjects were explicitly asked to not react to the flash immediately but to continue to pursue the target and to execute a saccade to the perceived flash position (PFP) after the pursuit target had disappeared. At the end of the pursuit, subjects saccaded to and fixated the perceived position of the flash. Then they pressed a button which initiated the display of a bright luminance stimulus. This stimulus, which prevented dark adaptation, had a difference of Gaussian (“Mexican hat”) luminance profile with a dark center, surrounded by a brighter area which faded out, covering almost the entire screen. It thus had no sharp edges in order to minimize retinal after-images. This stimulus stayed on the screen until the subject signaled readiness for the next trial by pressing the button one more time. Subjects were asked to limit eye blinks to the duration of the dark-adaptation prevention screen and were informed that blinks occurring at other times would lead to the trial being discarded.

### Fixation paradigm

As mentioned in the Introduction, spatial perception during steady fixation is not accurate but rather shows idiosyncratic patterns of localization error across subjects. Accordingly, we mapped each subject’s localization performance also during fixation. To this end, we used a paradigm which was identical to the main experiment’s pursuit paradigm, except that the oculomotor target did not move but rather was stable at the straight-ahead position during the whole trial. Analogous to the main experiment, the target was blanked for 300 ms in half of the trials. It is important to note, though, that by this experimental design, starting positions for the localization saccades were different in fixation as compared to pursuit trials. Previous studies on monkeys have shown an influence of the saccade starting position on the landing points of saccades toward remembered targets (Barton and Sparks, 2001). Importantly, in our study we compared localization during pursuit with and without blanking and the baseline correction affected both pursuit conditions alike.

### Oculomotor behavior

Eye-movement data were analyzed using MATLAB ^®^ 2010a (The Mathworks Inc., Natick, MA, USA). Eye velocity was computed as the discrete derivative of the position data delivered by the EyeLink II system. Trials were excluded if blinks occurred during the pursuit phase of the experiment or if the button press occurred later than 5 s after the pursuit target had vanished since we considered this a loss of attentiveness. Furthermore, trials were excluded from further analysis if saccades occurred within 300 ms before to 300 ms after flash presentation to exclude effects of saccades on localization. Saccades were detected if the eye velocity of three consecutive samples differed more than 20°/s from the average value of the preceding 50 ms. For the fixation paradigm a total of about 1,700 trials or, respectively, 20% of all trials (minimum: 6% for Subject 1; maximum: 31% for Subject 3; median: 22%) were discarded based on the above described criteria. For the pursuit paradigm the total number of discarded trials was about 9,000 or 44% (minimum: 18% for Subject 4; maximum: 53% for Subject 3; median: 47%).

### Perceived flash position (PFP)

Subjects were instructed to saccade to and fixate the point of perceived flash position (PFP) after the pursuit target was extinguished. Once fixating the PFP they pressed a button. We chose not to use the eye position at the time of the button press as estimate of the PFP because subjects often started blinking in anticipation of the upcoming bright screen before they had actually pressed the button. In addition, inspection of eye position traces revealed prolonged drifts during fixation in darkness which could confound exact determination of the PFP. Thus, the PFP was defined as the end point of a “localization saccade” or the end point of a “correction saccade” following the “localization saccade” that was detected most recently before the button press. For valid localization saccades, the endpoint of the first correction saccade was logged as PFP if its latency was less than 200 ms with respect to the end of the localization saccade and if its amplitude was smaller or equal to that of the localization saccade. We chose to include correction saccades, as these corrections could not be error corrections relative to external references (complete darkness) and thus had to be corrections relative to an internal target representation, i.e., the PFP. The validity of this approach was substantiated by the fact that the distribution of the PFPs for each subject for each of the conditions had less variance (higher precision) when taking into account corrections compared to using solely the endpoint of the initial saccade.

Double saccades with less than 25 ms inter-saccadic interval were merged into a single saccade since the final end point was of key interest to our analysis. Since the localization target (flash) was presented 2° below the pursuit trajectory, we used the vertical amplitude to distinguish the “localization saccade” from catch-up saccades during pursuit. Finally, PFP determination was only deemed valid if the “localization saccade” occurred no earlier than 50 ms after pursuit target offset and if a fixation period of at least 300 ms followed the localization or, respectively, the correction saccade.

### Stimulus position and baseline correction

In our analysis we determined localization as a function of the retinal eccentricity of the flashed targets. To this end, in all four eye movement conditions [(i) fixation, with fixation target on; (ii) fixation, with fixation target blanked (gap fixation); (iii) steady-stated pursuit, with pursuit target on; (iv) steady-state pursuit, with pursuit target blanked (gap pursuit)] we determined the position of the flash, that had to be localized, with respect to the current eye position and not with respect to the current position of the (visible or invisible) fixation or pursuit target on a trial by trial basis. In a first step we evaluated the perceived flash position for each subject in the fixation and the pursuit paradigm. To this end we calculated the localization error as *perceived minus presented target position.* Calculations always considered only the horizontal components of the presented and the perceived stimulus position (see above: PFP).

Based on previous work, we expected localization during fixation to not be accurate. It has been established in the literature to functionally characterize localization biases as centripetal or centrifugal (Kaminiarz et al., 2007; Kaminiarz et al., 2008; Willeke et al., 2022). In case of a centripetal bias, stimulus locations are perceived closer toward straight-ahead than they physically are. Likewise, a centrifugal bias indicates that stimulus positions are perceived further away from straight ahead than they physically are. High resolution mapping of foveal and parafoveal spatial perception has revealed a centrifugal bias for small target eccentricities (<about 4°) and a centripetal bias for larger target eccentricities (>about 4°; Willeke et al., 2022). Furthermore, localization error during fixation has been shown to be idiosyncratic, influencing also localization during eye movements (Kaminiarz et al., 2007, 2008). In these studies, the authors had investigated spatial perception during OKAN. Initially, raw data appeared non-consistent across participants. However, when the observed spatial perception during OKAN was corrected for localization during fixation at a single subject level, results became consistent for the group of observers. Consequently, to identify the net effect of pursuit on localization in the current study, we subtracted the observed localization error during fixation from the observed error during pursuit (baseline correction). This baseline correction was performed individually for each subject, visibility condition [gap (open-loop) vs. continuous (closed-loop)] and flash position.

Our experimental paradigm introduced a delay of 1,200 ms between flash presentation and the go-signal for the saccade (i.e., extinguishing the fixation or pursuit target). Hence, flash position had to be held in memory and localization errors in principle could be related to this memory period and possible memory decay. There are two important points to consider, though. First, this memory period was identical in the two trial conditions that we focused on in this study, i.e., those with and without blanking of the (fixation or pursuit) target. Second, this delay is a standard approach in studies on localization in the context of eye movements [e.g., Lappe et al., 2000; Kaminiarz et al., 2007; Kaminiarz et al., 2008; Burr et al., 2011; Bremmer et al., 2017; recently reviewed in Binda and Morrone (2018)]. In addition to the temporal offset, in the pursuit trials, the eyes were at about 12° in the periphery, when the saccade was triggered. It is known from the literature that eccentric eye positions can have an impact on saccades to remembered targets (Barton and Sparks, 2001). While these results come from the trained macaque monkey, there is good reason to assume that such orbital effects would also occur in human observers. Yet, like for the temporal offset, the spatial offset at the end of the pursuit was identical for pursuit with and without target blanking. Hence, if existent, it would have affected performance in both pursuit conditions alike.

#### Gap related drop in velocity

Occluding the pursuit target during smooth pursuit leads to a drop in eye velocity (Becker and Fuchs, 1985). In order to determine the start of the gap- induced drop in eye velocity we adopted a procedure previously established to determine the time of pursuit onset after initial fixation (Schütz et al., 2007; Blanke et al., 2010). This procedure uses two linear fits to two different epochs of pursuit and searches for the time-point of intersection of these two linear fits. In analogy to this approach in the context of pursuit onset, in our study the first regression was applied to a pre-gap period of steady-state pursuit. The pre-gap regression was calculated for the 500 ms preceding the start of target occlusion (T_Start_ = −700 to T_End_ = −200 ms). For the within-gap regression we used a sliding time window of 75 ms width to find the regression having the steepest slope. The onset of the gap induced decrease in eye velocity was identified as the point in time of intersection of these two regression functions. The end of the gap-related decay in eye velocity was determined for each subject as the time of the minimum of the low pass filtered eye velocity (symmetrical 2nd order Butterworth filter, 5 Hz cut-off frequency) within the gap period.

## Results

### Localization during fixation

**FIGURE 2:**
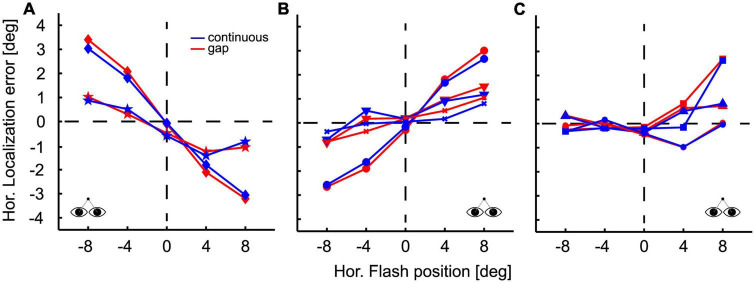
Localization during fixation. Flash position is plotted on the abscissa, localization error on the ordinate, defined as perceived minus presented flash position. Positive error values indicate that the PFP was to the right of the flash; negative error values indicate a perceptual shift to the left. Accordingly, a centripetal bias of flash localization is indicated by positive error values for stimuli presented in the left visual field and negative values for stimuli presented in the right visual fields. Likewise, a centrifugal bias is characterized by negative error values for stimuli in the left visual field and positive error values for stimuli in the right visual field. Conditions are color-coded: results for the continuous target presentation are depicted in blue and those for the gap condition in red. Different symbols indicate data from different subjects. Localization results during fixation were quite different across participants. Data are grouped for illustration purposes. Two subjects exhibited a centripetal bias **(A)** three subjects showed a centrifugal bias **(B)** and three subjects exhibited differences in localization in the left and right hemifield **(C)**. Within subjects, differences in localization between pursuit conditions (continuous vs. gap) were not significant, except for one subject for one single target location (+4°) **(C)**.

During fixation the perceived location of the flash was not accurate. Figure 2 shows the localization performance in the fixation paradigm, both, for the continuous (blue lines and symbols) and the gap (red lines and symbols) condition, for all subjects (*n* = 8). Localization performance was quite different across participants and could be classified into three distinct groups (Figures A–C). In all three panels we show localization error defined as *perceived minus presented stimulus location*. Two of the participants perceived stimulus locations closer to the midline (or straight-ahead, SA) than they were presented physically (Figure 2A), which is called a *centripetal* bias. In such case, localization error is positive for stimuli in the left visual field and negative for stimuli in the right visual field. As an example, one of the participants showed a strong centripetal bias (diamond shaped symbols): this participant perceived stimuli presented at −8°, i.e., 8° in the left visual field, at about −5°, resulting in a localization error of +3°. Likewise, stimuli presented at +8°, i.e., 8° in the right visual field, were perceived at 5°, resulting in a localization error of −3°. The other participant revealed a comparatively small centripetal bias (star shaped symbols) of roughly 1° for stimuli at 8° eccentricity. Three subjects (Figure 2B) showed a centrifugal bias: they generally perceived flashes as being more eccentric than they physically were. The behaviors of the remaining three subjects are shown in Figure 2C. These subjects showed differences for the perception of flashes presented in the left and right hemifield. Target occlusion had hardly any influence on localization during fixation, except for one subject for one single target location (+4, Figure 2C). A 5 (flash positions) × 2 (visibility condition) ANOVA on subjects’ mean localization error revealed no significant main effects or interactions (*p* > 0.05).

### Localization during smooth pursuit

**FIGURE 3:**
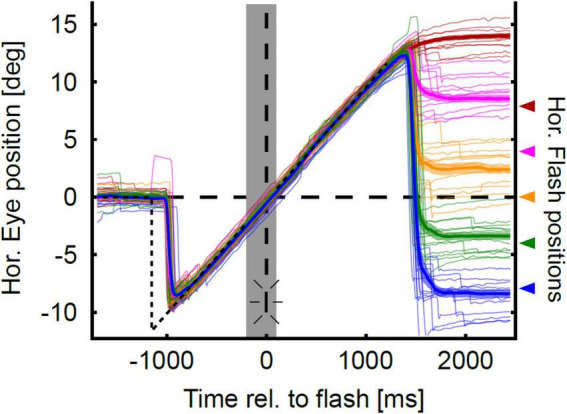
Eye position traces in the gap-condition. Sample horizontal eye position traces of one representative subject are color-coded for each flash position (horizontal at −8°, −4°, 0°, 4°, 8°) and aligned to flash onset. The thick colored lines show mean eye position over time, and the lighter colored trace around the mean eye position indicates the confidence intervals as determined by bootstrapping. Time of flash onset is indicated by the vertical dashed line and the flash symbol. Color-code corresponds to the horizontal flash positions which are indicated with arrows at the right axis of the figure. The gray shaded area indicates the duration of target blanking. The horizontal position trace of the pursuit target is indicated by the dashed black line. Eye position traces are initially on target. Target step occurs at −1,200 ms. With a delay of about 240 ms the eyes catch up the moving target and closely follow it despite target blanking. After a latency of about 150 ms after target extinction (at 1,200 ms), the subject executed eye movements toward the perceived horizontal position of the flash. Localization errors can be coarsely estimated as the distance between the mean eye trace and the corresponding flash position.

Figure 3 shows the localization of a representative subject in the gap-pursuit paradigm. At the end of each pursuit trial the subject made an eye movement toward the perceived flash position (PFP). Real target positions are shown at the right ordinate and are color-coded. The eye position after the localization saccades (see section “Materials and methods”) differed clearly for the different target positions. Yet, localization was not accurate. Localization errors were rather small for the two flash positions in the hemifield contralateral to pursuit direction (left hemifield, rightward pursuit). For the central target position the error was larger and further increased for the two flash positions in the ipsilateral hemifield.

**FIGURE 4:**
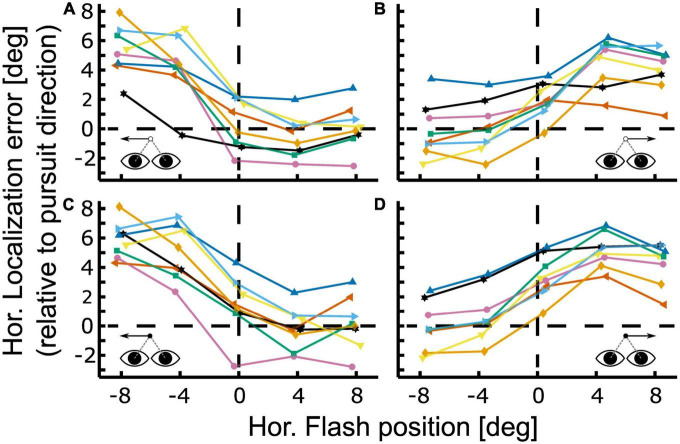
Localization during open- and closed-loop pursuit. Localization error is shown for the gap [panels **(A,B)**] and continuous condition [panels **(C,D)**] for leftward [panels **(A,C)**] and rightward [panels **(B,D)**] Pursuit. Colored lines with symbols depict baseline-corrected single subject data. Positive values indicate localization errors in pursuit direction. All cases show the typical smooth pursuit localization error pattern with small errors in the contraversive hemifield (the hemifield the eyes come from) and larger errors in the ipsiversive hemifield (the hemifield into which the eyes move). The representative subject shown in Figure 3 corresponds to the green line with square markers in this figure.

Baseline corrected results for localization of this participant in the four different conditions [leftward vs. rightward pursuit; steady-state (closed-loop) vs. gap (open-loop)] are shown in Figure 4 in an eye-centered representation. Localization error is depicted relative to pursuit direction, i.e., *positive localization error* indicates that the localization target (flash) was perceived further ahead of its real physical position in the direction of the pursuit. All four conditions show the typical smooth pursuit localization error pattern with small errors in the contraversive hemifield (the hemifield the eyes come from) and larger errors in the ipsiversive hemifield (the hemifield into which the eyes move). Maximum localization error for leftward pursuit was found at the most eccentric flash position, i.e., at −8° (Figures 4A, C). Here, the average error was 5.33 ± 0.60° (population mean ± SE) for the gap condition and 5.85 ± 0.43° for the steady-state condition. For rightward pursuit the largest localization error for both visibility conditions was found at +4°: 4.46 ± 0.58° in the gap-condition and 5.17 ± 0.41° in the continuous condition (Figures 4B, D). Pairwise comparisons of localization error between flash positions did not reveal significant differences for comparisons within the same hemifield (−8 vs. −4 and +4 vs. +8°) for equal pursuit directions and visibility conditions (continuous vs. gap).

In a next step we performed a 5 (flash positions) × 2 (visibility condition; gap/steady-state) × 2 (pursuit directions; left-/rightward) ANOVA on the baseline corrected localization error results of the individual subjects. We found a significant main effect of target visibility [F (1,159) = 5.2, *p* < 0.05] and flash position [F (4,159) = 67.88, *p* < 0.01] and a significant interaction between flash position and pursuit direction [F (4,159) = 4.38, *p* < 0.01]. Tukey’s HSD *post-hoc* test revealed that localization error was significantly stronger for continuous than for gap-pursuit (*p* < 0.05). On average, localization error was 23% smaller in the gap-condition.

**FIGURE 5:**
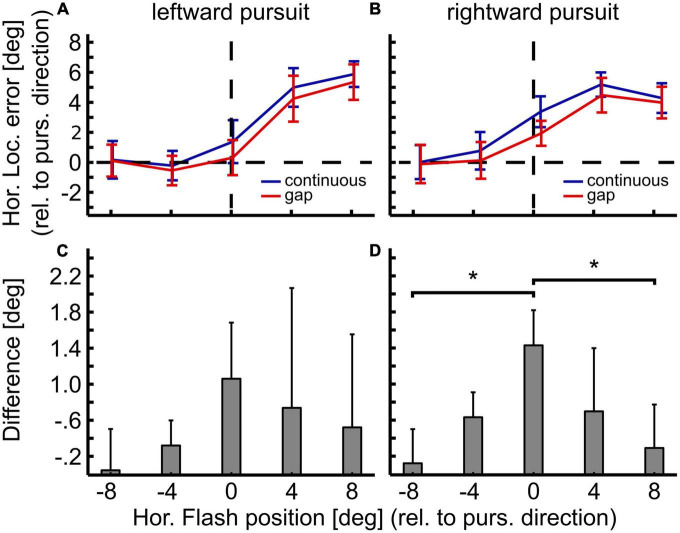
Differences in localization between open- and closed-loop condition. Panels in the top row show mean localization error and 95%-confidence intervals for gap (open-loop, red) and continuous (closed-loop, blue) conditions for leftward **(A)** and rightward pursuit **(B)**. Panels in the bottom row show the difference in localization error between both conditions for leftward **(C)** and rightward pursuit **(D)** with 95%-confidence intervals. Overall, localization error was slightly smaller for the gap condition. Yet, this reduction was spatially specific: It was strongest for the straight-ahead flash position and smallest in the periphery. These spatial differences, however, were only statistically significant between the central and the most eccentric flash positions (±8°) for rightward pursuit. *p < 0.05, Wilcoxon signed–rank test, Bonferroni–corrected.

Figures 5A, B show the population values of the localization errors for leftward and rightward pursuit. Values on the abscissa indicate flash positions relative to pursuit direction. Positive values indicate flash positions ahead of the pursuit target, i.e., in the direction of the pursuit. Negative values indicate flash position behind the pursuit target, i.e., opposite to pursuit direction. The differences between the visibility conditions (continuous vs. gap) were largest for the straight-ahead position and smaller toward the periphery and not related to the localization error magnitude (Figures 5C, D). *Post hoc* comparison of the localization error between continuous and gap pursuit revealed a significant difference only for the straight-ahead position during rightward pursuit (Figures 5B, D, Wilcoxon signed-rank test, *p* < 0.05, Bonferroni-corrected) for which the difference between conditions was 1.43° or 42%. The results for leftward pursuit showed the same error pattern, yet, the differences between central visual field and periphery were not statistically significant (*p* > 0.1).

### Localization: The role of eye velocity

**FIGURE 6:**
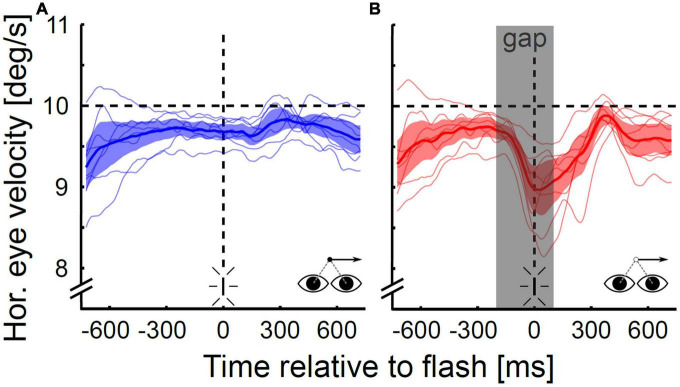
Horizontal eye velocity during open- and closed-loop pursuit. Data are shown for the continuous **(A)** and the gap condition **(B)** for rightward pursuit for the population of subjects (*N* = 8). The gray shaded area in **(B)** depicts the target occlusion duration (gap). On average, the eye velocity in the gap condition started to drop 112 ms after gap onset and was 0.7°/s or 7.3% lower at the time of the flash than in the steady-state condition. The minimum velocity was on average reached at 227 ms after gap onset after which subjects either maintained this reduced level for a short duration (∼150–300 ms) or started to re-accelerate.

Figure 6 shows the mean eye velocities for all subjects individually (thin lines) and the population mean averaged across subjects (thick lines with 95%-CI) for both visibility conditions. Pooled across directions, the subjects’ mean gain (eye velocity divided by target velocity) at the time of the flash differed significantly between the continuous and the gap condition (Wilcoxon signed rank test, *p* < 0.05). The subject’s mean gain varied between 0.94 and 0.99 (population mean: 0.96) in the continuous condition and between 0.84 and 0.96 (population mean: 0.89) in the gap condition. On average, the subjects’ gap-related decrease in eye velocity started between 97 and 132 ms (population mean: 112 ms) after target occlusion. For all subjects the end of the gap-related drop in eye velocity occurred before the target reappeared: it was observed between 176 and 288 ms (population mean: 227 ms) after target occlusion. In five of eight subjects, the end of the gap-related drop in eye velocity was followed by re-acceleration; the other three subjects maintained a residual velocity for a short time (∼150–300 ms) before starting to re-accelerate.

**FIGURE 7:**
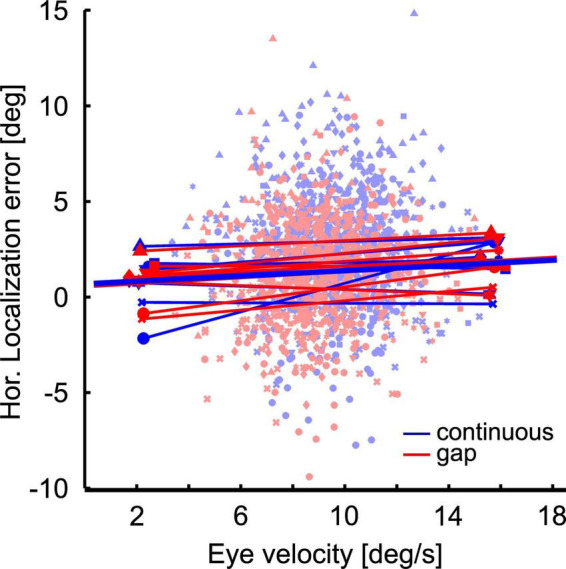
Localization error as a function of eye velocity. The scatter plot shows at the single trial level the localization error as function of eye velocity at the time of flash presentation at straight ahead position. Colors (red and blue) indicate the conditions: Continuous (blue) vs. gap (red). Different symbols depict data from different subjects. Thin straight lines are regressions of the individual data sets, thick lines are the averages of these regressions.

Localization error and eye velocity generally had smaller values in the gap as compared to the continuous condition. This could be indicative of a correlation or even a causal relationship between the two values. In order to determine whether or not there is evidence for such a relationship, we performed a correlation analysis. Since *post hoc* analysis revealed a significant difference in localization behavior between continuous and gap-pursuit only for the straight-ahead flash position for rightward pursuit we limited our analysis to this case. We correlated each subjects’ localization error for the straight-ahead position in the two visibility conditions (16 correlations; eight subjects * two visibility conditions) with eye velocity at the time of stimulus presentation. Our analysis did not reveal any significant correlations: correlation coefficients ranged from −0.04 to 0.15, with a mean value of 0.03. For illustration purposes Figure 7 shows linear regressions for the 16 cases color coded by visibility condition (red for the gap condition and blue for the continuous condition). The population mean regressions had a slope of m_*SST*_ = 0.018 and m_*Gap*_ = 0.045 s. We used these slope values to determine the amount of change in localization error induced by the mean velocity difference at the time of the flash between the continuous and the gap condition (0.7°/s). The above-mentioned values predicted a localization difference of 0.02°, which was only about 1.4% of the observed difference. In other words: even though eye velocity at the time of the flash was generally smaller in the gap condition than in the continuous condition, our data do not provide evidence for the idea that the reduction of localization error in the gap condition was due to this reduced eye velocity.

## Discussion

Previous studies have shown flashed targets to be mislocalized during different forms of slow eye-movements, i.e., pursuit initiation (Blanke et al., 2010), steady-state pursuit (Mateeff et al., 1981; van Beers et al., 2001; Rotman et al., 2004; Kerzel et al., 2006; Koenigs and Bremmer, 2010), OKN (Kaminiarz et al., 2007; Tozzi et al., 2007) and OKAN (Kaminiarz et al., 2008). OKAN, the open-loop continuation of OKN, elicits a dramatically different localization pattern than the other smooth eye movements. In our current study, we investigated localization performance during open-loop smooth pursuit in order to determine whether putative localization errors would be pursuit-like or OKAN-like. Open-loop pursuit was induced by temporarily occluding the pursuit target (gap). We investigated localization during this open-loop pursuit (gap condition) and compared it with localization during closed-loop pursuit (continuous condition) in the same participants. On average, localization error was smaller in the gap condition than in the continuous condition. Localization errors in both visibility conditions were generally in the direction of pursuit and larger in the hemifield the eye traveled toward (ipsilateral hemifield) than in its wake (contralateral hemifield). Hence, localization during open-loop pursuit is clearly pursuit-like.

### Behavioral paradigm

In our paradigm we decided to employ saccades in order to probe the participants’ spatial perception. Several aspects have to be considered in this context. First, after flash presentation, pursuit continued for roughly 1,200 ms. Hence, saccades toward the perceived flash location were memory guided. This delay in response could have affected saccade performance e.g., accuracy or precision. Yet, a localization response only after the end of the ongoing eye movements is a standard approach in the field [e.g., Lappe et al., 2000; Kaminiarz et al., 2007; Kaminiarz et al., 2008; Burr et al., 2011; Bremmer et al., 2017; recently reviewed by Binda and Morrone (2018)] and makes our study comparable with others. Furthermore, a possible influence of the delay on saccade performance would have affected pursuit with and without blanking alike. In addition, one might ask if saccades at all are suited to measure spatial perception (Lisi and Cavanagh, 2015). In that study, Lisi and Cavanagh reported a dissociation between the perceptual and saccadic localization of moving objects. It has to be mentioned, though, that the authors took advantage of a perceptual illusion in which visual motion signals presented within the boundaries of a peripheral moving object can make the object’s apparent trajectory deviate by 45° or more from its physical trajectory. Further processing steps might have contributed to their finding of a dissociation of spatial perception and action. Instead, in our study we assumed a shared representation of visual and saccadic motor space, a view shared by others [recently reviewed by Zimmermann and Lappe (2016)].

### Localization during closed-loop and open-loop pursuit

The reduction of localization error during open-loop pursuit as compared to closed-loop pursuit was not evenly distributed in (retinal) space. Rather, the largest difference in localization error between open-loop and closed-loop pursuit was found for the straight-ahead position. In contrast, the influence of target occlusion on localization error was smallest in the periphery (±8°). If the reductive effect of target occlusion had been related to the overall magnitude of localization error as previously determined during steady-state pursuit, we would have expected large differences in the ipsiversive periphery, small differences in the contraversive periphery, and an intermediate value at the straight-ahead position. Our findings are clearly different and suggest that target occlusion has a spatially specific effect on localization during smooth pursuit. This effect could occur with respect to the eyes or the head (or body or world). Since in our experiments, eyes, head and body were always directed straight ahead at the time of the flash, we cannot dissociate between the different reference frames. Furthermore, it has been demonstrated that the latency of localization saccades as well as the smooth eye velocity have an influence on the residual error (Blohm et al., 2003; Blohm et al., 2005). Thus, any localization error reported in our experiments is likely due to the combination of two factors: (1) a bias in the interpretation of retinal flash information due to the eye movement (Blohm et al., 2003) and (2) an error in the integration of extra-retinal information (Dowiasch et al., 2016). Additional experiments would be needed to tackle these aspects.

### The neural correlate of localization error

The visual environment during open-loop pursuit and OKAN are identical (*absence* of any visual stimulus) and clearly different from closed-loop pursuit (*presence* of a foveal visual target). Nevertheless, localization error during open-loop pursuit is pursuit-like rather than OKAN-like. This suggests that it is not the visual input signal that causes localization errors during smooth eye movements.

Previous studies have shown that both closed-loop and open-loop smooth pursuit as well as OKN share a largely overlapping neural circuitry and are cortically driven (Bremmer et al., 2002; Schlack et al., 2003; Trillenberg et al., 2004; Konen et al., 2005; Nagel et al., 2006; Schraa-Tam et al., 2008; Kashou et al., 2010; Kleiser et al., 2017). For all three types of slow eye movements localization error is in the direction of the slow eye movement. During OKAN, however, no influence of eye-movement direction on localization error was found (Kaminiarz et al., 2008). Studies in the macaque suggest that OKAN is not accompanied by specific cortical activity at all (Ilg, 1997). While, to our best knowledge, comparable data on humans are not available, we consider it most likely that also human OKAN is purely subcortically driven. This could imply that the lack of cortical activation might be the key difference distinguishing OKAN localization from localization during both closed-loop and open-loop pursuit and OKN.

Subjects were able to smoothly track the moving target even when it was blanked for 300 ms. As expected, subjects’ eye velocity decayed during the occlusion phase. As previously described by Becker and Fuchs (1985) the decay of velocity was not complete but rather stabilized at a residual velocity. Some subjects even re-accelerated their eye movements after reaching minimum velocity, which could be an indicator to a prediction effect, since the gap was always 300 ms long and the pursuit trajectory was predictable. The reduction in eye velocity, however, did not correlate with the differences in target localization between the two pursuit conditions. Accordingly, eye velocity does not seem to be tightly linked to localization error during smooth eye movements.

This leaves us with the question about the neural basis of localization during open-loop pursuit. We suggest various, probably independent processes to be at work. As no foveal visual target exists during open loop pursuit, pursuit localization might be governed by the internal representation of the target or eye position. For instance, a recent study by Dowiasch et al. (2016) showed that a constant lead of the decoded eye position signal recorded in the brain in combination with a common attentional bias ahead of the pursuit target (Khan et al., 2010) described the localization error pattern of briefly flashed targets during smooth pursuit very well. As a second mechanism, the spatial proximity of the flash with respect to the pursuit target might exhibit an additional influence. Mateeff and colleagues had shown that the size of localization error in the direction of pursuit decreased with higher stimulus intensity (Mateeff et al., 1982). One could argue that the perceived intensity of the flash at the straight-ahead position was higher in total darkness (gap-pursuit) than during visually guided pursuit. This difference in perceived intensity would explain the specific reduced localization error during open-loop pursuit. Finally, the two pursuit conditions might have been accompanied by different attentional states. It is well-known that a flashing light attracts involuntary attention (Remington et al., 1992). Accordingly, the re-appearance of the blanked target might have attracted the subjects’ attention. This effect would have been largest close to the position of the invisible target, i.e., at the straight-ahead position.

## Data availability statement

The raw data supporting the conclusions of this article will be made available by the authors, without undue reservation.

## Ethics statement

The studies involving human participants were reviewed and approved by Ethikkomission FB Psychologie, Philipps-Universität Marburg, Fb. 04 - Psychologie, Gutenbergstraße 18, 35032 Marburg (AZ-2012-23K). The participants provided their written informed consent to participate in this study.

## Author contributions

SD, JK, and FB contributed to conception and design of the study. MB performed the measurement and statistical analysis. SD and MB wrote the manuscript. All authors contributed to manuscript revision, read, and approved the submitted version.
